# Gender differences in the association between healthy eating index-2015 and hypertension in the US population: evidence from NHANES 1999–2018

**DOI:** 10.1186/s12889-023-17625-0

**Published:** 2024-01-31

**Authors:** Jiayi Weng, Yukang Mao, Qiyang Xie, Kangyun Sun, Xiangqing Kong

**Affiliations:** 1 Department of Cardiology, The Affiliated Suzhou Hospital of Nanjing Medical University, Suzhou Municipal Hospital, Gusu School, Nanjing Medical University, Suzhou, 215008 China; 2 Department of Cardiology, The First Affiliated Hospital of Nanjing Medical University, Nanjing, 210029 China

**Keywords:** NHANES, HEI-2015, Hypertension, Gender difference, Cross-sectional study

## Abstract

**Background:**

Diet has long been recognized as an important modifiable risk factor for hypertension. Herein, our research goal was to decipher the association of healthy eating index-2015 (HEI-2015) with hypertension, and to explore potential gender differences.

**Methods:**

We collected the cross-sectional data of 42,391 participants of the National Health and Nutrition Examination Survey (NHANES) 1999–2018. The association of HEI-2015 with hypertension was estimated using weighted multivariate logistic regression, with restricted cubic spline (RCS) regression being adopted to examine the nonlinearity of this association in both genders, and the stability of the results were examined by sensitivity analysis. We also performed subgroup analysis to detect potential difference in the link between HEI-2015 and hypertension stratified by several confounding factors.

**Results:**

After eliminating potential confounding bias, the adjusted odds ratios (ORs) with 95% confidence intervals (CIs) for hypertension across higher HEI-2015 quartiles were 0.93 (0.85–1.03), 0.84 (0.77–0.93), and 0.78 (0.72–0.86) compared to the lowest quartile, respectively. HEI-2015 was nonlinearly and inversely associated with hypertension in all participants. The gender-specific RCS curves presented a U-shaped correlation in males, while showed a linear and inverse correlation in females. Besides, subgroup analyses showed a lower risk of hypertension in participants who were females, younger than 40 years, Whites, obese, and diabetic patients.

**Conclusions:**

We determined a nonlinear and inverse association between HEI-2015 and hypertension in the US general population, and revealed a remarkable gender difference when adhering to a HEI-2015 diet for preventing hypertension.

**Supplementary Information:**

The online version contains supplementary material available at 10.1186/s12889-023-17625-0.

## Introduction

Contemporarily, it is generally accepted that hypertension along with related cardio-cerebrovascular complications are world-class killers, jeopardizing the quality of life of hundreds of thousands of people, causing immense socio-economic burden, and posing an unprecedented challenge to human health [1]. As evidenced by a pooled analysis of 1201 population-based studies, the global number of people aged 30–79 years with hypertension — defined as anyone whose systolic/diastolic blood pressure (SBP/DBP) exceeds 140/90 mmHg, or those regularly taking anti-hypertensive medications — has grown dramatically during the past few decades, rising from 648 million in 1990 to 1278 million in 2019 [2]. Compared to developed countries, a pronouncedly higher prevalence of hypertension is observed in underdeveloped and developing countries [3], which may be largely attributed to unawareness of home blood pressure monitoring [4] and the lack of timely and effective pharmacological interventions [5]. Hypertension can be clinically categorized into two major types: essential hypertension (hypertension without an identifiable cause) and secondary hypertension (hypertension related to a specific cause), the former of which is a complex and multifactorial disorder wherein multiple genetic, behavioral, and environmental factors together contribute to elevated systemic blood pressure, perpetuate hypertensive status, and ultimately give rise to severe and irreversible damage to vital organs, and accounts for over 90% of hypertensive cases worldwide [6]. Among these risk factors, undesirable lifestyles, poor eating habits in particular, are most likely to be in direct contact with the general population and exert detrimental effects on the cardiovascular system; therefore, lifestyle adjustment represents an indispensable step towards maintaining normal blood pressure at present.

Diet is one of the most important aspects of lifestyle interventions for preventing and treating hypertension, predominantly owing to the modifiable nature of this factor and its close relations with other risk factors for hypertension (e.g., diabetes, obesity, and hyperlipidemia). There has been solid epidemiological evidence that a poor-quality diet is strongly related to an increased morbidity and mortality of cardiovascular diseases (CVDs), including hypertension [7]. Instead of comprising single foods or nutrients, a specific dietary pattern is generally a composite of diverse food ingredients; in this context, the Health Eating Index (HEI) was proposed by the US Department of Agriculture (USDA) to estimate an individual’s overall dietary quality, specifically the extent to which a dietary pattern is in conformity with the Dietary Guidelines for Americans (DGA), and was updated every five years to accommodate each new edition of the DGA [8]. Since the release of the first edition of HEI (HEI-2005), it was employed for the assessment of the quality of the diet consumed by the US residents [9] and soon spread to several countries without any adjustment to the scoring system, leading to a surge of publications in which HEI was applied as a useful tool to investigate diet-disease relation [10]. More recently, this index has further evolved into a universal criterion for detecting the disparities in dietary quality based on the mainstream eating habits of 185 countries [11]. In this study, we chose the most up-to-date version of the HEI (HEI-2015) [12], which has been proven to have satisfactory validity and reliability for assessing the impact of dietary quality on the risk of hypertension [13]. Hitherto, several epidemiological studies that investigated the association between HEI and hypertension have implied that consumption of a diet with a higher HEI score is closely related to a reduced risk of hypertension [14–21], whereas such inverse association has not yet been validated in a nationally representative sample of non-institutionalized civilians in the US; therefore, the primary goal of this cross-sectional study was to detect the association of HEI-2015 with hypertension in the general US population via analyzing data from the National Health and Nutrition Examination Surveys (NHANES).

## Methods

### Study population and ethics

**Fig. 1:**
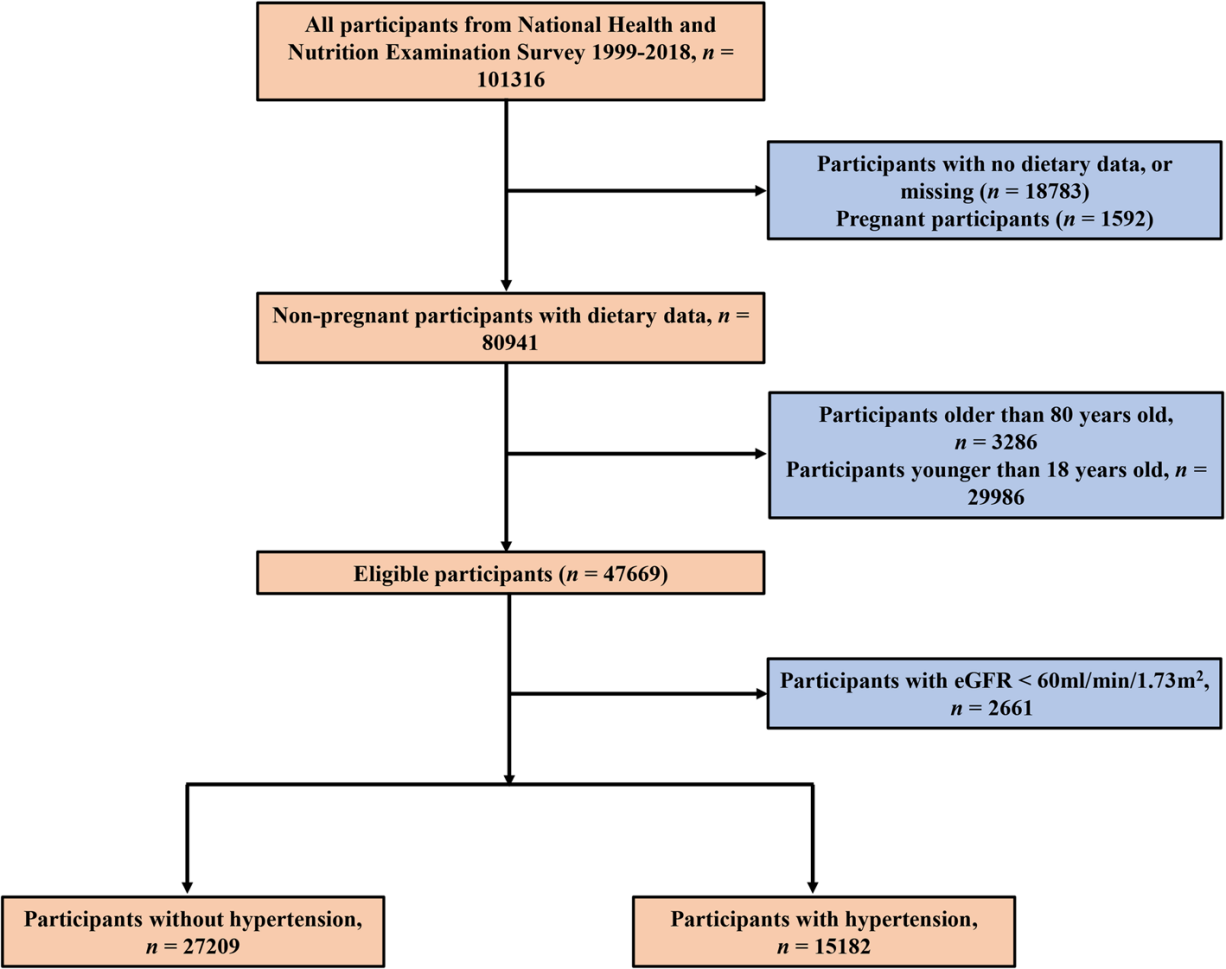
A detailed flow chart of participant recruitment

NHANES is a nationwide campaign launched by National Center for Health Statistics (NCHS) that mainly focuses on the health and nutritional condition of the noninstitutionalized US civilians at two-year intervals, and whose aim is to obtain a comprehensive knowledge of contemporary disease profiles and to provide references for formulating public health policies. All of the NHANES data is accessible to the public and can be downloaded freely through: https://www.cdc.gov/nchs/nhanes/index.htm. In this study, cross-sectional data of 101,316 participants from ten consecutive cycles of the NHANES (1999–2018) were initially included. The exclusion criteria were set as our previous studies using NHANES database to exploring risk factors of hypertension: (1) participants aged < 18 or ≥ 80 years (*n* = 33,272); (2) participants who were pregnant (*n* = 1,592); (3) participants without relevant information on dietary intake or hypertension (*n* = 18,783); (4) participants whose estimated glomerular filtration rate (eGFR) < 60 ml/min/1.73m^2^ (*n* = 2,661) [22–24]. After manual data filtration, we ultimately selected a total of 42,391 participants for subsequent analyses. The study protocol has gained approval from the NHANES Institutional Review Board, with informed consent being obtained from all the participants. A detailed flow chart of study participant recruitment was presented in Fig. 1.

### Assessment of dietary quality

Since NHANES surveys are a series of large-scale population-based campaigns, it is necessary to collect dietary information rapidly and conveniently. To this end, 24-h dietary data — including the types and amounts of food and drinks consumed during the 24-h period prior to the interview in the mobile examination center (MEC) — was collected by trained interviewers following a standardized protocol, and was then used to calculate the HEI-2015 score as previously reported [23, 25]. According to the 2015–2020 DGA, a total of 13 dietary components taken into account in the HEI-2015 scoring system can be divided into two categories, one of which comprises nine adequacy components that are encouraged to be adequately consumed (Total Fruits, Whole Fruits, Total Vegetables, Greens and Beans, Whole Grains, Dairy, Total Protein Foods, Seafood and Plant Proteins, and Fatty Acids), and the other one of which is composed of four moderation components, the recommended intake of which should be strictly confined within a modest range (Refined Grains, Sodium, Added Sugars, and Saturated Fats). Each dietary component is assigned a maximal score of 5 or 10, which was then summed up to generate an overall HEI-2015 score, the theoretical reference range of which is 0–100. A dietary pattern advocating for higher intake of adequacy components as well as lower intake of moderation components usually corresponds to a higher HEI-2015 score, which is commonly indicative of a better quality of the diet [12].

### Assessment of hypertension

Hypertension can be defined based on either self-reported previous diagnosis by a physician or blood pressure measured during physical examination. A standardized procedure recommended by American Heart Association was conducted for blood pressure measurements. After sitting in a comfortable position for at least 5 min, three single measurements were performed by well-trained clinicians equipped with mercury sphygmomanometers at half-minute intervals. To mitigate accidental errors caused by blood pressure fluctuations, the mean value of all three readings was calculated and recorded as one participant’s blood pressure. Anyone who met at least one of the criteria listed below was considered as having hypertension: (1) Average systolic blood pressure (SBP) ≥ 140 mmHg; (2) Average diastolic blood pressure (DBP) ≥ 90 mmHg; (3) Self-reported diagnosis of hypertension; (4) Current use of anti-hypertensive medications [26]. More relevant information about blood pressure measurement is available on the NHANES website (http://www.cdc.gov/nchs/data/nhanes/pe.pdf). To be noted, use of self-reported measures are prone to recall bias, which may have an impact on the interpretation of the data.

### Covariates

Being based on previous publications and biological considerations, we collected as much covariates with known confounding effects on hypertension as possible. Demographic features including age, sex, race/ethnicity, educational level, smoking status, and alcohol consumption were obtained by standardized questionnaires and face-to-face interviews. Physical examination and laboratory tests were performed by experienced medical workers step by step in the MEC.

Race/ethnicity were divided into five categories: non-Hispanic White, non-Hispanic Black, other Hispanic, Mexican American, and other races. The following educational levels were included: below high school, high school, and above high school. Participants who smoked over 100 cigarettes throughout their lifetime were defined as smokers, regardless of whether he/she had quitted smoking at the time of interview [27], and those consuming at least 12 drinks during the year preceding the survey were considered alcohol drinkers [28]. Body mass index (BMI), calculated as weight in kilograms (kg) divided by the square of height in meters (m^2^), is widely used for estimating overweight/obesity status. A BMI score greater than 25 and 30 is recognized as the major diagnostic criteria of overweight and obesity in clinical practice, respectively [29]. Fasting blood glucose (FBG) and serum concentrations of glycated hemoglobin (HbA1c), triglyceride (TG), total cholesterol (TC), low-density lipoprotein cholesterol (LDL-C), high-density lipoprotein cholesterol (HDL-C) were examined by standardized laboratory tests. For calculating eGFR, NHANES investigators applied a formula developed by the Chronic Kidney Disease Epidemiology Collaboration (CKD-EPI) in which variables including age, sex, race/ethnicity, and serum creatinine (SCr) were incorporated to adapt to different populations [30].

Besides, diabetes was another important confounder that may have an impact on hypertension and other cardiovascular diseases [31, 32]. Anyone who provided a previous diagnosis of diabetes by a physician or health professional was defined as patients with diagnosed diabetes, while those without diagnosed diabetes but with a HbA1c level 6.5% (47.5 mmol/mol) or higher, FPG level 126 mg/dL (7.0 mmol/L) or higher, or 2-h oral glucose tolerance test (OGTT) plasma glucose 200 mg/dL or higher (11.1 mmol/L) tested by laboratory examinations were classified as having undiagnosed diabetes. Participants with diagnosed diabetes or undiagnosed diabetes were both considered diabetic patients [33, 34].

### Statistical analysis

Since NHANES survey employed a series of complex sampling designs, we took into account the sample weights corresponding to different research periods in our analytic methods to yield accurate estimates of health-related statistics [35–38]. Continuous variables were presented in the form of weighted mean and standard deviation (SD), whereas categorical variables were expressed as frequencies and percentages. For the purpose of detecting differences in baseline characteristics between participants with and without hypertension, continuous and categorical variables were compared using student's t-test and chi-square test, respectively. The HEI-2015 score was categorized into four quartiles (Q1: HEI-2015 < 40.3; Q2: 40.3 ≤ HEI-2015 < 49.6; Q3: 49.6 ≤ HEI-2015 < 58.2; Q4: HEI-2015 ≥ 58.2), with the first quartile (Q1) being the reference quartile.

We used a variety of multivariate logistic regression models to estimate the odds ratios (ORs) and 95% confidence intervals (CIs) for detecting the association of HEI-2015 with hypertension. Adjustments for age and study circle was performed in Model I; Model II was further adjusted for sex and race/ethnicity; Model III was adjusted for additional confounders including educational level, smoking status, alcohol consumption, diabetes, and eGFR in addition to those adjusted in Model II. We also applied restricted cubic spline (RCS) regression with 3 knots (10th, 50th, and 90th percentiles) to examine the nonlinearity of the association between HEI-2015 and hypertension. Subgroup analyses in terms of age, sex, race/ethnicity, BMI, diabetes were conducted to verify whether the association between HEI-2015 and hypertension remained stable across different subgroups. A sensitivity analysis was also performed to validate the stability of the association between HEI-2015 and hypertension. R software version 4.1.6 (http://www.R-project.org, The R Foundation, Vienna, Austria) was used for all statistical analyses, and a two-tailed *P*-value < 0.05 was considered statistically significant.

## Results

### Baseline characteristics of the study participants

Detailed information about the baseline characteristics of all participants grouped by hypertensive status was illustrated in Table 1. In brief, 42,391 participants were enrolled in the analysis, with a weighted average age of 43.88 years. The overall prevalence of hypertension among all participants was 35.81%, and the weighted mean HEI-2015 score (95% CI) was 49.93 (49.57–50.28). Compared to those without hypertension, hypertensive individuals tended to be older, males, non-Hispanic White/Black, less educated, non-smokers, and diabetic patients, and had higher levels of FBG, HbA1c, TG, TC, and LDL-C as well as lower levels of HDL-C and eGFR (all *P* < 0.001), while no statistical significant difference was observed in alcohol consumption between two groups (*P* = 0.73). Of note, among all participants with hypertension, 9809 participants (64.6%) were taking antihypertensive drugs. Moreover, the blood pressure of 6963 participants (71.0%) was controlled within the normal range among all participants taking antihypertensive drugs. Overall, the mean HEI-2015 score of hypertension group was slighter higher than that of non-hypertension group (50.46 vs. 49.66, *P* < 0.001); therefore, we subsequently investigated the divergence in the HEI-2015 score of each dietary component between two groups, and found higher scores of Total Vegetables, Total Fruits, Whole Fruits, Whole Grains, Total Protein Foods, Fatty Acids, Refined Grains, and Added Sugars and lower scores of Dairy, Sodium, and Saturated Fatty Acids (all *P* < 0.05), with the exception of Greens and Beans (*P* = 0.17) and Seafood and Plant Proteins (*P* = 0.1), in participants with hypertension rather than those without hypertension (Table 2). Besides, Supplementary Table 1 and Supplementary Table 2 presented baseline characteristics of the study participants and several cardiometabolic indexes grouped by HEI-2015 quartiles, respectively.

**Table 1:** Baseline characteristics of the study participants grouped by hypertensive status

**Variables**	**Overall** (***n* = 42,391**)	**Non-hypertension** (***n* = 27,209**)	**Hypertension** (***n* = 15,182)**	***P* value**
Age, years	43.88(43.53,44.23)	39.29(38.92,39.66)	53.17(52.79,53.55)	< 0.001***
Sex-male, %	49.96(48.19,51.73)	49.08(48.43,49.73)	51.74(50.75,52.73)	< 0.001***
Race, %				< 0.001***
Non-Hispanic White	67.98(63.88,72.07)	67.25(65.23,69.27)	69.45(67.16,71.74)	
Non-Hispanic Black	10.70(9.77,11.64)	9.38(8.43,10.32)	13.38(11.88,14.89)	
Mexican American	8.74(7.73,9.74)	10.01(8.80,11.21)	6.16(5.16,7.15)	
Other Hispanic	5.85(5.01,6.70)	6.38(5.43,7.33)	4.79(3.97,5.61)	
Other	6.73(6.18,7.29)	6.98(6.36,7.60)	6.22(5.56,6.89)	
Smoking, %	21.98(20.87,23.09)	23.37(22.36,24.37)	20.98(20.06,21.90)	< 0.001***
Drinking, %	82.08(78.98,85.19)	89.30(88.32,90.29)	89.16(88.21,90.10)	0.73
Educational level, %				< 0.001***
Below high school	5.10(4.71,5.49)	4.57(4.19,4.96)	6.19(5.55,6.84)	
High school	36.02(34.22,37.82)	34.97(33.62,36.33)	38.21(36.87,39.54)	
Above high school	58.81(56.37,61.25)	60.45(58.95,61.96)	55.60(54.11,57.09)	
SBP, mmHg	120.70(120.38,121.02)	114.57(114.33,114.80)	133.06(132.56,133.56)	< 0.001***
DBP, mmHg	71.52(71.22,71.81)	69.31(69.03,69.59)	75.97(75.56,76.39)	< 0.001***
Diabetes, %	10.82(10.28,11.36)	5.43(5.05,5.80)	21.76(20.89,22.63)	< 0.001***
FBG, mmol/L	5.78(5.75,5.81)	5.53(5.50,5.56)	6.28(6.21,6.34)	< 0.001***
HbA1c, %	5.53(5.51,5.54)	5.38(5.37,5.40)	5.82(5.79,5.84)	< 0.001***
eGFR, ml/min/1.73m^2^	98.55(98.11,98.99)	101.99(101.49,102.49)	91.58(91.15,92.02)	< 0.001***
TG, mmol/L	1.47(1.45,1.50)	1.36(1.32,1.39)	1.71(1.66,1.75)	< 0.001***
TC, mmol/L	5.05(5.04,5.07)	4.98(4.96,5.00)	5.20(5.17,5.23)	< 0.001***
LDL-C, mmol/L	2.99(2.97,3.01)	2.97(2.95,2.99)	3.04(3.01,3.07)	< 0.001***
HDL-C, mmol/L	1.37(1.36,1.37)	1.38(1.37,1.39)	1.34(1.33,1.35)	< 0.001***

Continuous variables are presented as weighted mean [95% CI], and categorical variables are presented as unweighted frequencies or percentages [95% CI]. SBP, systolic blood pressure; DBP, diastolic blood pressure; FBG, fasting blood glucose; HbA1c, glycated hemoglobin; eGFR, estimated glomerular filtration rate; TG, triglyceride; TC, total cholesterol; LDL-C, low-density lipoprotein cholesterol; HDL-C, high-density lipoprotein cholesterol. *** *P* value < 0.001

**Table 2:** Comparison of total HEI-2015 and each dietary component-specific HEI-2015 between participants with and without hypertension

Variables	Overall (*n* = 42,391)	Non-hypertension (*n* = 27,209)	Hypertension (*n* = 15,182)	*P* value
HEI	49.93(49.57,50.28)	49.66(49.26,50.07)	50.46(50.07,50.85)	< 0.001***
Total Vegetables	3.02(2.99,3.05)	2.98(2.95,3.01)	3.10(3.06,3.15)	< 0.001***
Greens and Beans	1.45(1.41,1.49)	1.46(1.42,1.50)	1.42(1.37,1.48)	0.17
Total Fruits	2.00(1.95,2.05)	1.97(1.92,2.02)	2.06(2.00,2.11)	0.002**
Whole Fruits	2.04(1.99,2.09)	2.00(1.95,2.06)	2.13(2.07,2.19)	< 0.001***
Whole Grains	2.19(2.13,2.25)	2.14(2.06,2.21)	2.30(2.22,2.37)	< 0.001***
Dairy	4.98(4.91,5.04)	5.07(5.00,5.13)	4.79(4.71,4.88)	< 0.001***
Total Protein Foods	4.17(4.15,4.19)	4.13(4.11,4.16)	4.24(4.21,4.26)	< 0.001***
Seafood and Plant Proteins	2.23(2.19,2.27)	2.22(2.17,2.26)	2.27(2.21,2.33)	0.1
Fatty Acids	4.93(4.86,4.99)	4.88(4.80,4.95)	5.03(4.94,5.11)	0.003**
Sodium	4.61(4.55,4.67)	4.69(4.62,4.76)	4.45(4.38,4.53)	< 0.001***
Refined Grains	6.04(5.98,6.10)	5.97(5.90,6.04)	6.18(6.10,6.26)	< 0.001***
Saturated Fats	5.93(5.87,6.00)	5.97(5.90,6.04)	5.86(5.77,5.95)	0.02*
Added Sugars	6.33(6.25,6.42)	6.19(6.09,6.28)	6.63(6.53,6.73)	< 0.001***

Data of total HEI-2015 and each dietary component-specific HEI-2015 are presented as weighted mean (95% CI). CI, confidence interval; HEI-2015, healthy eating index-2015. * *P* value < 0.05, ** *P* value < 0.01, *** *P* value < 0.001

### Association of HEI-2015 with hypertension

**Fig. 2:**
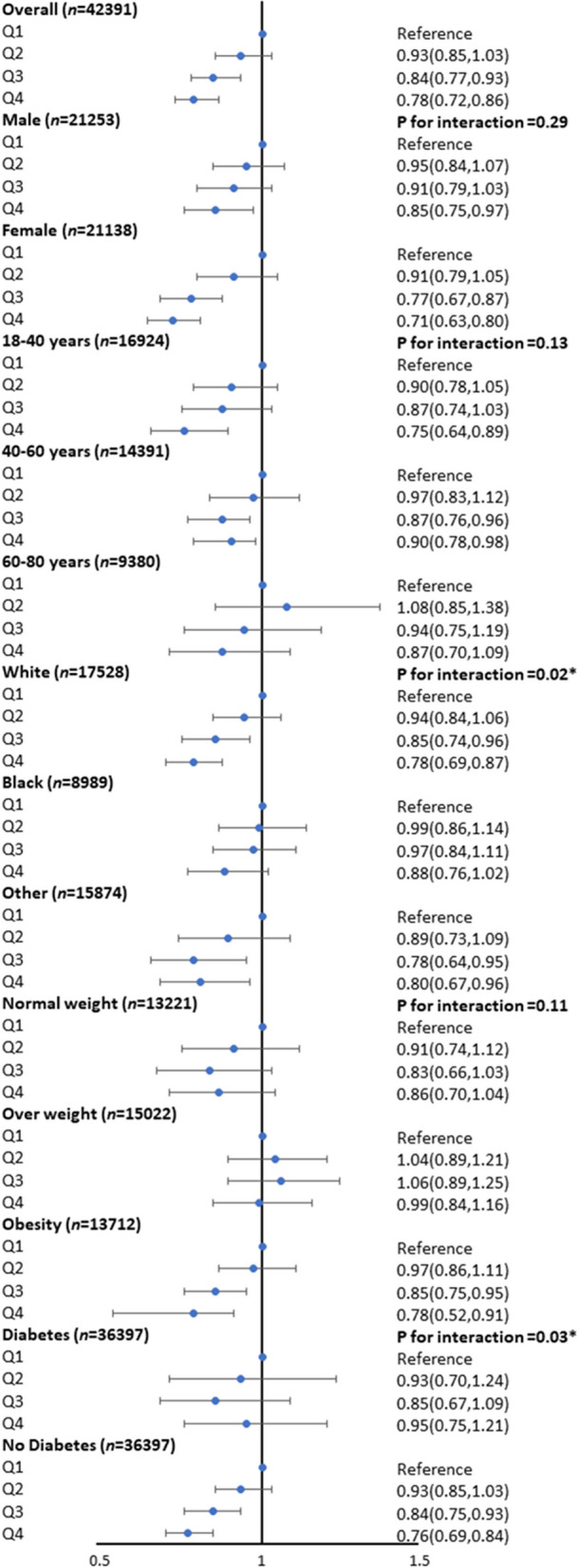
**A** The RCS curve of the association between HEI-2015 and hypertension among all the study participants. RCS regression was adjusted for age, sex, race/ethnicity, study circle, educational level, smoking status, alcohol consumption, diabetes, and eGFR. **B** The RCS curves of the associations between HEI-2015 and hypertension among female (colored red) and male participants (colored blue), respectively. RCS, restricted cubic spline; HEI-2015, healthy eating index-2015; BMI, body mass index; OR, odds ratio

We performed a sampling-weighted multivariate logistic regression analysis for detecting the association between HEI-2015 and hypertension, and revealed that a higher HEI-2015 was correlated with a lower risk of hypertension, which remained consistent across all three regression models. After adjustment of potential confounders, the adjusted ORs with 95% CIs for hypertension in increasing quartiles of HEI-2015 were 0.93 (0.85–1.03), 0.84 (0.77–0.93), and 0.78 (0.72–0.86) compared to the lowest quartile, respectively (Table 3). As shown in Fig. 2A, RCS curve displayed a nonlinear and inverse association of HEI-2015 with hypertension in all participants. We also explored whether gender affects the association of HEI-2015 with the risk of hypertension. In males, a U-shaped correlation between HEI-2015 and hypertension was observed, with the increase in HEI-2015 score appearing to be associated with a reduced risk of hypertension on the left part of the RCS curve and being associated with an increased odds of developing hypertension on its right part. In contrast, a linear and inverse dose–response relation between HEI-2015 and hypertension was found in females (Fig. 2B).

**Table 3:** Weighted logistic regression analysis on the association between HEI-2015 and hypertension

	**Model I**		**Model II**		**Model III**	
	**OR [95% CI]**	***P* value**	**OR [95% CI]**	***P* value**	**OR [95% CI]**	***P* value**
**Q1**	Reference	-	Reference	-	Reference	-
**Q2**	0.90(0.83,0.98)	0.01*	0.90(0.83,0.98)	0.01*	0.93(0.85,1.03)	0.15
**Q3**	0.80(0.73,0.87)	< 0.001***	0.81(0.74,0.88)	< 0.001***	0.84(0.77,0.93)	< 0.001***
**Q4**	0.70(0.65,0.76)	< 0.001***	0.72(0.66,0.78)	< 0.001***	0.78(0.72,0.86)	< 0.001***

Data are presented as OR [95% CI]. Model I was adjusted for age and study circle; Model II was adjusted for age, sex, race/ethnicity, and study circle; Model III was adjusted for age, sex, race/ethnicity, study circle, educational level, smoking status, alcohol consumption, diabetes, and eGFR. HEI-2015, healthy eating index-2015; OR, odds ratio; CI, confidence interval. * *P* value < 0.05, *** *P* value < 0.001

### Subgroup analysis

**Fig. 3:**
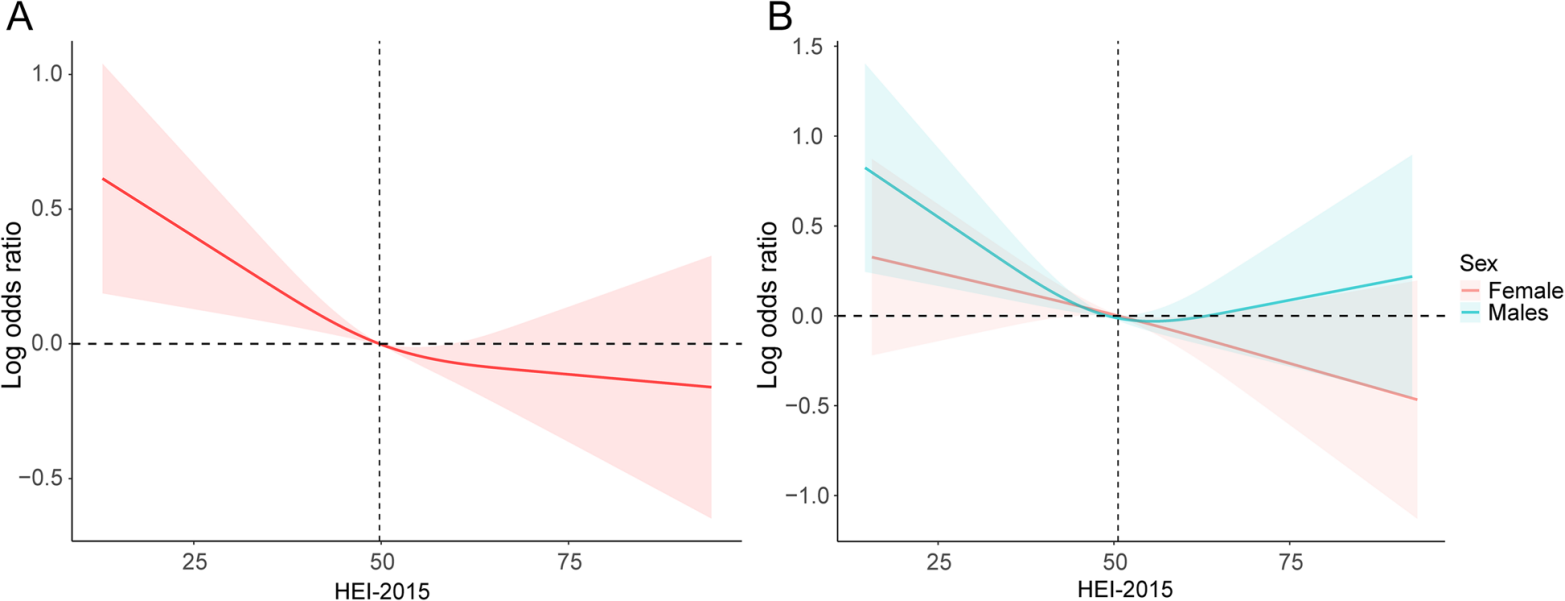
Subgroups analyses for the association between HEI-2015 and hypertension. Analyses were adjusted for sex (male or female), age (≤ 40 years, 40–60 years, or ≥ 60 years), race/ethnicity (Black, White, or others), BMI (normal weight, overweight, or obesity), and diabetes (yes or no), and hypertension (yes and no). HEI-2015, healthy eating index-2015; BMI, body mass index

Subgroup analyses stratified by sex (male or female), age (≤ 40 years, 40–60 years, or ≥ 60 years), race/ethnicity (Black, White, or others), BMI (normal weight, overweight, or obesity), and diabetes (yes or no) were performed to explore whether the association between HEI-2015 and hypertension remained stable among populations with diverse subgroup characteristics. According to the forest plot, we found significant interactions for the association between HEI-2015 and hypertension in subgroups divided by race/ethnicity (*P* for interaction = 0.02) and diabetes (*P* for interaction = 0.03). A more pronounced inverse association of HEI-2015 with hypertension can be observed in participants who were females, younger than 40 years, Whites, obese, and diabetic patients, with increasing HEI-2015 quartiles displaying a trend towards lower odds of hypertension, implying that these individuals were inclined to be exempt from developing hypertension (Fig. 3).

### Sensitivity analysis

In line with the findings of weighted logistic regression analysis, an inverse association of HEI-2015 with hypertension was also determined by sensitivity analysis, and consistently noted in all adjusted models. In the fully adjusted model (Model III), the odds of having hypertension decreased with advancing HEI-2015 score (Q2: 0.97 (0.90–1.04); Q3: 0.87 (0.81–0.93); Q4: 0.84 (0.78–0.90)), suggesting that participants in higher quartiles of HEI-2015 were less likely to be susceptible to hypertension (Table 4). Overall, sensitivity analysis confirmed the stability and reliability of the results generated by sampling-weighted logistical regression analysis.

**Table 4:** Unweighted logistic regression analysis on the association between HEI-2015 and hypertension in sensitivity analysis

	**Model I**		**Model II**		**Model III**	
	**OR [95% CI]**	***P* value**	**OR [95% CI]**	***P* value**	**OR [95% CI]**	***P* value**
**Q1**	Reference	-	Reference	-	Reference	-
**Q2**	0.93(0.87,0.99)	0.03*	0.93(0.88,1.00)	0.04*	0.97(0.90,1.04)	0.33
**Q3**	0.81(0.76,0.87)	< 0.001***	0.83(0.78,0.89)	< 0.001***	0.87(0.81,0.93)	< 0.001***
**Q4**	0.75(0.70,0.80)	< 0.001***	0.78(0.73,0.84)	< 0.001***	0.84(0.78,0.90)	< 0.001***

Data are presented as OR [95% CI]. Model I was adjusted for age and study circle; Model II was adjusted for age, sex, race/ethnicity, and study circle; Model III was adjusted for age, sex, race/ethnicity, study circle, educational level, smoking status, alcohol consumption, diabetes, and eGFR. HEI-2015, healthy eating index-2015; OR, odds ratio; CI, confidence interval. * *P* value < 0.05, *** *P* value < 0.001

## Discussion

According to the present analysis, our primary finding was a nonlinear and inverse association of HEI-2015 with hypertension independently of multiple confounding factors in the US general population through analyzing the cross-sectional data of 42,391 NHANES participants, with such association being further proven to be more pronounced among participants who were females, younger than 40 years, Whites, obese, and diabetic patients, suggesting that these individuals were less likely to predispose to hypertension. It is also noteworthy that a remarkable gender difference was found in the association between HEI-2015 and hypertension. Compared to earlier studies in which the inverse link between HEI and hypertension was confirmed, a prominent advantage of our research is that all discoveries were established upon the NHANES database, which is a nationwide survey that recruits non-institutionalized US residents from the entire country in an unprejudiced manner, performs standardized experimental procedures, and utilizes rigorous analytic methods for data processing and quality control, guaranteeing the authenticity and reliability of the data to the maximum extent.

Dietary intake, as one of the most important modifiable risk factors that affect cardiovascular health, has been well-documented to exert a double-edged effect on blood pressure. A poor dietary quality is generally accepted as a precursor to hypertension, while a good recipe may be promising to produce substantial cardiovascular benefits [39]. When estimating the impact of different dietary patterns with varying dietary qualities on the prevalence of hypertension with HEI, almost all previous studies consistently supported the notion that a higher HEI score was indicative of a reduced odds of developing hypertension, which was completely in line with our findings. According to a randomized controlled trial (RCT), hypercholesterolemic patients who were randomly assigned to a dietary intervention for cardiovascular fitness had a larger degree of increase in HEI-2005 score ((5.5 (2.7–8.4) vs. 0.1 (-4.3–4.6)) as well as a larger degree of decrease in blood pressure (SBP: -6.5 mmHg; 95% CI: -10.3 to -2.7 mmHg vs. -5.4 mmHg; 95% CI: -11.4 to -0.7 mmHg; DBP: -3.4 mmHg; 95% CI: -6.6 to -0.008 mmHg vs. -2.2 mmHg; 95% CI: -7.7 to 2.9 mmHg, respectively) than those assigned to the control group over a 3-month follow-up [14]. In a cross-sectional study based on a large-scale cohort of healthy individuals residing in central Italy, Bendinelli et al. found a significantly inverse association of HEI-2010 with blood pressure level, with the reduction in SBP/DBP values becoming more evident with increasing adherence to HEI-2010 [15]. Through analyzing the cross-sectional data of 2,459 African American adolescents (aged 12–21 years) enrolled in the 2005–2016 cycles of NHANES, Ducharme-Smith and colleagues demonstrated that participants in the highest HEI-2010 quartile had 0.81 (95% CI: 0.53–1.24) times the odds of developing pediatric hypertension (1–12 years: SBP/DBP ≥ 95th percentiles for those aged 1–12 years; ≥ 13 years: SBP/DBP ≥ 130/80 mmHg) compared to those in the lowest quartile [16], which was supported by another Iranian population-based cross-sectional study in which the risk of hypertension decreased across increasing HEI-2015 quartiles in fully adjusted logistic regression model (Q2: 0.91 (0.79–1.04); Q3: 0.80 (0.70–0.91); Q4: 0.79 (0.68–0.90)) [17]. Apart from cross-sectional studies, similar conclusions can also be drawn from longitudinal studies. In the Ravansar non-communicable diseases (RaNCD) cohort study conducted in Iran, individuals in the top quartile of HEI-2015 had a 39% and 30% reduction of incident hypertension risk in comparison to their counterparts in the bottom quartile in the crude (0.61 (0.46–0.82)) and fully adjusted model (0.70 (0.51–0.97)), respectively [18]. Another study based on the 1946–1951 birth cohort of the Australian Longitudinal Study on Women’s Health (ALSWH) revealed that women who adhere to HEI-2010 more strictly were far less vulnerable to hypertension [19]. Furthermore, an inverse correlation between HEI and hypertension was also observed in women who experienced diabetes during pregnancy [20], or even pregnant ones [21]. Intriguingly, despite the heterogeneity of study populations, involving divergence in gender, age, country/region, race/ethnicity, and so on, as well as the fact that dietary quality was evaluated based on different editions of the HEI scoring system in these studies and ours (e.g., HEI-2005, HEI-2010, HEI-2015), the association of HEI with hypertension remains highly consistent across different studies, which seemingly reflects that HEI is a universally applicable approach for predicting the risk of hypertension based on dietary information. Mechanistically, better adherence to HEI-based diet has been proven to be favorable for lowering levels of biomarkers of inflammation and oxidative stress [40], and the intimate link between an increase in HEI and a decrease in dietary inflammatory index (DII), an index that was designed for assessing the inflammatory potential of a specific dietary pattern and was higher proportionally with rising pro-inflammatory capacity of a diet, has also been confirmed [25, 41]. Given the pivotal roles of systemic inflammation and oxidative stress in eliciting elevated blood pressure [42], the beneficial effects of HEI-based dietary patterns in preventing hypertension may be partially attributed to suppression of these processes.

A serendipitous finding drawn from the RCS regression is that the association between HEI-2015 and hypertension among US adults differs by gender, which was not reported by previous research. In the RCS curve for males, an increase in HEI-2015 is accompanied by a proportionally decrease in hypertension risk on the left side of an inflection point located at a HEI-2015 score of around 50, something that causes a sudden shift towards an opposite trend on the right side of the inflection point, meaning that any further increase in dietary quality unexpectedly contributes to higher susceptibility to hypertension at this point. As for females, the odds of developing hypertension drops at a constant speed as HEI-2015 score climbs, which ultimately gives rise to a linear and inverse dose–response relation between the two. Given that gonadal hormones are indispensable players in causing gender differences in physiology and disease and that estrogen is a kind of feminine hormone capable of protecting against hypertension as evidenced by a wealth of medical literature [43, 44], it could make sense as to why hypertension risk is always lower among females versus males, while the relevant evidence on whether the observable disparities in the association between HEI-2015 and hypertension is attributable to hormonal differences is sparse at present. Taken together, although the explicit mechanisms for this gender difference is not entirely clear, current evidence provided by our research appears to imply that pursuing a high-quality diet mindlessly may not always be a desirable option for preventing hypertension among male residents in the US; therefore, any decision on choosing a dietary pattern theoretically believed to be healthier should be made prudently so as to ensure maximal physical benefits and avoid unwanted effects. On the flip side, better adherence to HEI-2015 diet is more meaningful for mitigating the risk of hypertension in females.

Our study has several shortcomings that need to be noted and addressed in further research: (1) Due to the inherent limitations of cross-sectional design, it remains a tough task to infer causality between HEI-2015 and hypertension, which requires to be further validated in more longitudinal studies with prospective cohorts of US adults, especially those studies on male and female cohorts, respectively; (2) Although we have attempted to screen as much covariates as possible to control for confounding bias, given hypertension is a complex and multifactorial disorder that involves multiple genetic, behavioral, and environmental etiologies, there may still exist some unknown or unidentified confounders that may also have a role in the pathogenesis and progression of hypertension as they are not explicitly documented in NHANES database; (3) Technically, a single 24-h recall is perhaps not the best method to calculate habitual dietary intake at an individual level because of the unneglectable day-to-day variations in dietary intake, whereas the sheer amounts of participants included in the NHANES surveys may preclude the practical application of some better options (e.g., multiple 24-h recalls, food frequency questionnaire (FFQ)) in deciphering the long-term link between dietary intake and hypertension, which should be addressed in further studies using NHANES dietary information [45]; (4) In most contemporary population-based surveys, including NHANES, a diagnosis of hypertension relies on an average value of several blood pressure measurements obtained during a single visit, which may predictably give rise to an overestimate of hypertension prevalence compared with what would be found by using an average value of ≥ 2 readings taken on ≥ 2 visits, as recommended in current and previous clinical practice guidelines [46, 47]; (5) Since self-reporting is one of the most useful means to conveniently and rapidly obtain relevant information about dietary intake and how frequently hypertension occurs among NHANES participants, whereas such an approach may inevitably lead to recall bias owing to the restrictions of self-reported methods. Thus, caution should be taken during the analysis and interpretation of the data; (6) Hypertension is simply generalized as a single disease entity rather than classified into different types (essential hypertension or secondary hypertension) in the NHANES database; however, heterogeneity in the etiology and pathophysiological feature of different types of hypertension may serve as important causes of the potential differences in the association between HEI-2015 and hypertension among different individuals. In other words, whether the link between HEI-2015 and hypertension remains stable among individuals with different types of hypertension requires to be elucidated by future studies.

## Conclusion

Collectively, we found a nonlinear and inverse association between HEI-2015 and hypertension independently of potential confounding factors in the US general population, and revealed a remarkable gender difference in this association. Given the inherent limitations of cross-sectional research, further studies are warranted to verify the causality of this association, decipher the underlying mechanisms whereby gender affects the link between HEI-2015 and hypertension, and formulate a more persuasive answer to the question of whether adhering to a healthier diet has equivalent efficacy for preventing hypertension.

### Supplementary Information

**Additional file 1: Supplementary Table 1.** Baseline characteristics of the study participants grouped by HEI-2015 quartiles.

**Additional file 2: Supplementary Table 2. **Measurements of cardiometabolic indexes grouped by HEI-2015 quartiles.

## Data Availability

All data analyzed in the current study are freely accessible on the NHANES website (https://www.cdc.gov/nchs/nhanes/index.htm).

## Abbreviations

BMI: Body mass index
CI: Confidence interval
CKD-EPI: Chronic Kidney Disease Epidemiology Collaboration
CVD: Cardiovascular disease
DASH: Dietary Approaches to Stop Hypertension
DBP: Diastolic blood pressure
DGA: Dietary Guidelines for Americans
DII: Dietary inflammatory index
eGFR: Estimated glomerular filtration rate
FBG: Fasting blood glucose
HbA1c: Glycated hemoglobin
HDL-C: High-density lipoprotein cholesterol
HEI: Healthy eating index
LDL-C: Low-density lipoprotein cholesterol
MEC: Mobile examination center
NCHS: National Center for Health Statistics
NHANES: National Health and Nutrition Examination Survey
OGTT: Oral glucose tolerance test
OR: Odds ratio
PUFA: Polyunsaturated fatty acid
RCS: Restricted cubic spline
RCT: Randomized controlled trial
ROS: Reactive oxygen species
SBP: Systolic blood pressure
SCr: Serum creatinine
SD: Standard deviation
TC: Total cholesterol
TG: Triglyceride
USDA: US Department of Agriculture

## References

[1] Zhou B (2021). Global epidemiology, health burden and effective interventions for elevated blood pressure and hypertension. Nat Rev Cardiol.

[2] NCD Risk Factor Collaboration (NCD-RisC) (2021). Worldwide trends in hypertension prevalence and progress in treatment and control from 1990 to 2019: a pooled analysis of 1201 population-representative studies with 104 million participants. Lancet.

[3] Mills KT (2016). Global Disparities of Hypertension Prevalence and Control: A Systematic Analysis of Population-Based Studies From 90 Countries. Circulation.

[4] Chow CK (2013). Prevalence, awareness, treatment, and control of hypertension in rural and urban communities in high-, middle-, and low-income countries. JAMA.

[5] Attaei MW (2017). Availability and affordability of blood pressure-lowering medicines and the effect on blood pressure control in high-income, middle-income, and low-income countries: an analysis of the PURE study data. Lancet Public Health.

[6] Whelton PK (2018). 2017 ACC/AHA/AAPA/ABC/ACPM/AGS/APhA/ASH/ASPC/NMA/PCNA Guideline for the prevention, detection, evaluation, and management of high blood pressure in adults: executive summary: a report of the American College of Cardiology/American heart association task force on clinical practice guidelines. Circulation.

[7] Afshin A, et al. Health effects of dietary risks in 195 countries, 1990–2017: a systematic analysis for the Global Burden of Disease Study 2017. Lancet. 2019;393(10184):1958–72. 10.1016/S0140-6736(19)30041-8 PMC6899507 30954305

[8] Kennedy ET (1995). The Healthy Eating Index: design and applications. J Am Diet Assoc.

[9] Wilson MM, Reedy J, Krebs-Smith SM (2016). American Diet Quality: Where It Is, Where It Is Heading, and What It Could Be. J Acad Nutr Diet.

[10] Schap T, Kuczynski K, Hiza H (2017). Healthy Eating Index-Beyond the Score. J Acad Nutr Diet.

[11] Miller V (2022). Global dietary quality in 185 countries from 1990 to 2018 show wide differences by nation, age, education, and urbanicity. Nat Food.

[12] Krebs-Smith SM (2018). Update of the Healthy Eating Index: HEI-2015. J Acad Nutr Diet.

[13] Reedy J (2018). Evaluation of the Healthy Eating Index-2015. J Acad Nutr Diet.

[14] Petrogianni M (2013). A multicomponent lifestyle intervention produces favourable changes in diet quality and cardiometabolic risk indices in hypercholesterolaemic adults. J Hum Nutr Diet.

[15] Bendinelli B (2019). A priori dietary patterns and blood pressure in the EPIC Florence cohort: a cross-sectional study. Eur J Nutr.

[16] Ducharme-Smith K (2021). Higher Diet Quality in African-American Adolescents Is Associated with Lower Odds of Metabolic Syndrome: Evidence from the NHANES. J Nutr.

[17] Motamedi A (2021). Diet quality in relation to the risk of hypertension among Iranian adults: cross-sectional analysis of Fasa PERSIAN cohort study. Nutr J.

[18] Pasdar Y (2022). Healthy eating index 2015 and major dietary patterns in relation to incident hypertension; a prospective cohort study. BMC Public Health.

[19] Hlaing-Hlaing H, et al. Diet Quality and Incident Non-Communicable Disease in the 1946–1951 Cohort of the Australian Longitudinal Study on Women's Health. Int J Environ Res Public Health. 2021;18(21):11375. 10.3390/ijerph182111375 PMC8583022 34769892

[20] Li S (2016). Healthful Dietary Patterns and the Risk of Hypertension Among Women With a History of Gestational Diabetes Mellitus: A Prospective Cohort Study. Hypertension.

[21] Li M (2021). Healthy dietary patterns and common pregnancy complications: a prospective and longitudinal study. Am J Clin Nutr.

[22] Chen L (2023). Association of different obesity patterns with hypertension in US male adults: a cross-sectional study. Sci Rep.

[23] Wu LD (2022). Associations between novel anthropometric measures and the prevalence of hypertension among 45,853 adults: A cross-sectional study. Front Cardiovasc Med.

[24] Zhou N (2022). The dietary inflammatory index and its association with the prevalence of hypertension: A cross-sectional study. Front Immunol.

[25] Wu L, et al. Dietary Inflammatory Index and Its Association with the Prevalence of Coronary Heart Disease among 45,306 US Adults. Nutrients. 2022;14(21):4553. 10.3390/nu14214553 PMC9656485 36364813

[26] Muntner P (2020). Trends in Blood Pressure Control Among US Adults With Hypertension, 1999–2000 to 2017–2018. JAMA.

[27] Liu C (2023). Association between serum total testosterone levels and metabolic syndrome among adult women in the United States, NHANES 2011–2016. Front Endocrinol (Lausanne).

[28] Chen X (2016). The Associations of Plant Protein Intake With All-Cause Mortality in CKD. Am J Kidney Dis.

[29] Curry SJ (2018). Behavioral Weight Loss Interventions to Prevent Obesity-Related Morbidity and Mortality in Adults: US Preventive Services Task Force Recommendation Statement. JAMA.

[30] Levey AS (2009). A new equation to estimate glomerular filtration rate. Ann Intern Med.

[31] Wu LD (2022). Glucose fluctuation promotes cardiomyocyte apoptosis by triggering endoplasmic reticulum (ER) stress signaling pathway in vivo and in vitro. Bioengineered.

[32] Wang X (2023). The awareness and determinants of diabetic foot ulcer prevention among diabetic patients: Insights from NHANES (2011–2018). Preventive Medicine Reports.

[33] Cheng YJ (2019). Prevalence of Diabetes by Race and Ethnicity in the United States, 2011–2016. JAMA.

[34] Wu LD (2023). Estimated pulse wave velocity is associated with all-cause mortality and cardiovascular mortality among adults with diabetes. Front Cardiovasc Med.

[35] Johnson C.L (2013). National health and nutrition examination survey: analytic guidelines, 1999–2010. Vital Health Stat.

[36] Chen TC (2018). National Health and Nutrition Examination Survey: Estimation Procedures, 2011–2014. Vital Health Stat.

[37] Chen TC (2020). National Health and Nutrition Examination Survey, 2015–2018: Sample Design and Estimation Procedures. Vital Health Stat.

[38] Zhou J (2023). Prevalence of neutropenia in US residents: a population based analysis of NHANES 2011–2018. BMC Public Health.

[39] Aljuraiban GS, et al. The role of diet in the prevention of hypertension and management of blood pressure: An umbrella review of meta-analyses of interventional and observational studies. Adv Nutr. 2023;100123. 10.1016/j.advnut.2023.09.011 PMC10831905 37783307

[40] Aleksandrova K, Koelman L, Rodrigues CE (2021). Dietary patterns and biomarkers of oxidative stress and inflammation: A systematic review of observational and intervention studies. Redox Biol.

[41] Wirth MD (2016). Anti-inflammatory Dietary Inflammatory Index scores are associated with healthier scores on other dietary indices. Nutr Res.

[42] Guzik TJ, Touyz RM (2017). Oxidative Stress, Inflammation, and Vascular Aging in Hypertension. Hypertension.

[43] Khalil RA (2005). Sex hormones as potential modulators of vascular function in hypertension. Hypertension.

[44] Arnold AP (2017). Sex Hormones and Sex Chromosomes Cause Sex Differences in the Development of Cardiovascular Diseases. Arterioscler Thromb Vasc Biol.

[45] Ahluwalia N (2016). Update on NHANES Dietary Data: Focus on Collection, Release, Analytical Considerations, and Uses to Inform Public Policy. Adv Nutr.

[46] Whelton PK (2018). 2017 ACC/AHA/AAPA/ABC/ACPM/AGS/APhA/ASH/ASPC/NMA/PCNA Guideline for the Prevention, Detection, Evaluation, and Management of High Blood Pressure in Adults: A Report of the American College of Cardiology/American Heart Association Task Force on Clinical Practice Guidelines. Circulation.

[47] Muntner P (2019). Measurement of Blood Pressure in Humans: A Scientific Statement From the American Heart Association. Hypertension.

