# Supplementary material for: Gender differences in the association between healthy eating index-2015 and hypertension in the US population: evidence from NHANES 1999–2018
Source: BMC Public Health. 2024 Jan 31;24:330. doi: 10.1186/s12889-023-17625-0 (PMC10829399; doi:10.1186/s12889-023-17625-0)
Supplement: Supplementary file 1 — Additional file 1: Supplementary Table 1. Baseline characteristics of the study participants grouped by HEI-2015 quartiles. [file 12889_2023_17625_MOESM1_ESM.docx]

**Supplementary Table 1 Baseline characteristics of the study participants grouped by HEI-2015 quartiles**

| Variables | HEI-Q1 | HEI-Q2 | HEI-Q3 | HEI-Q4 | *P* value |
| --- | --- | --- | --- | --- | --- |
| Age, years | 39.89(39.52,40.26) | 42.68(42.22,43.14) | 44.51(44.01,45.01) | 48.10(47.56,48.64) | <0.001*** |
| Sex-male, % | 53.28(51.99,54.58) | 51.51(50.32,52.70) | 50.39(49.26,51.51) | 45.09(43.97,46.20) | <0.001*** |
| Race, % |  |  |  |  | <0.001*** |
| Non-Hispanic White | 68.93(66.57,71.29) | 67.44(65.01,69.87) | 66.66(64.34,68.97) | 68.75(66.74,70.77) |  |
| Non-Hispanic Black | 12.06(10.70,13.42) | 12.11(10.75,13.47) | 10.39(9.19,11.58) | 8.42(7.48,9.37) |  |
| Mexican American | 8.15(6.95,9.34) | 9.05(7.83,10.28) | 9.64(8.33,10.94) | 8.19(7.11,9.28) |  |
| Other Hispanic | 5.33(4.38,6.28) | 5.45(4.47,6.43) | 6.21(5.23,7.20) | 6.38(5.37,7.40) |  |
| Other | 5.52(4.81,6.24) | 5.94(5.27,6.62) | 7.10(6.32,7.88) | 8.25(7.40,9.09) |  |
| Smoking, % | 31.32(29.97,32.68) | 26.64(25.36,27.92) | 21.08(19.93,22.23) | 12.30(11.43,13.17) | <0.001*** |
| Drinking, % | 89.81(88.93,90.68) | 90.06(88.85,91.26) | 89.30(88.15,90.44) | 87.99(86.75,89.22) | 0.001** |
| Educational level, % |  |  |  |  | <0.001*** |
| Below high school | 4.53(3.96,5.10) | 5.25(4.69,5.80) | 5.57(5.01,6.13) | 5.10(4.54,5.66) |  |
| High school | 45.83(44.04,47.61) | 39.23(37.70,40.76) | 34.39(32.86,35.92) | 25.53(24.17,26.88) |  |
| Above high school | 49.64(47.84,51.44) | 55.52(53.84,57.20) | 60.03(58.39,61.68) | 69.37(67.80,70.94) |  |
| SBP, mmHg | 120.26(119.81,120.71) | 120.69(120.21,121.17) | 120.78(120.30,121.26) | 121.05(120.52,121.57) | 0.1 |
| DBP, mmHg | 71.79(71.37,72.20) | 71.64(71.26,72.02) | 71.57(71.20,71.94) | 71.10(70.72,71.49) | 0.01* |
| Diabetes, % | 9.87(9.17,10.57) | 10.54(9.89,11.19) | 10.87(10.09,11.65) | 11.92(11.13,12.70) | <0.001*** |
| eGFR, ml/min/1.73m^2^ | 101.48(100.93,102.02) | 99.71(99.15,100.26) | 98.00(97.37,98.63) | 95.28(94.68,95.87) | <0.001*** |

Continuous variables are presented as weighted mean [95% CI], and categorical variables are presented as unweighted frequencies or percentages [95% CI]. HEI-2015, healthy eating index-2015; SBP, systolic blood pressure; DBP, diastolic blood pressure; eGFR, estimated glomerular filtration rate. * *P* value <0.05, ** *P* value <0.01, *** *P* value <0.001
