# Supplementary material for: Gender differences in the association between healthy eating index-2015 and hypertension in the US population: evidence from NHANES 1999–2018
Source: BMC Public Health. 2024 Jan 31;24:330. doi: 10.1186/s12889-023-17625-0 (PMC10829399; doi:10.1186/s12889-023-17625-0)
Supplement: Supplementary file 2 — Additional file 2: Supplementary Table 2. Measurements of cardiometabolic indexes grouped by HEI-2015 quartiles. [file 12889_2023_17625_MOESM2_ESM.docx]

**Supplementary Table 2 Measurements of cardiometabolic indexes grouped by HEI-2015 quartiles**

| Variables | HEI-Q1 | HEI-Q2 | HEI-Q3 | HEI-Q4 | *P* value |
| --- | --- | --- | --- | --- | --- |
| FBG, mmol/L | 5.77(5.72,5.81) | 5.79(5.74,5.84) | 5.79(5.74,5.84) | 5.78(5.72,5.84) | 0.93 |
| FBI, pmol/L | 83.09(80.15,86.03) | 78.25(75.64,80.86) | 76.22(72.91,79.53) | 67.10(64.84,69.36) | <0.001*** |
| HOMA-IR | 3.80(3.64,3.97) | 3.60(3.44,3.76) | 3.51(3.32,3.69) | 3.11(2.97,3.25) | <0.001*** |
| HbA1c, % | 5.51(5.49,5.53) | 5.53(5.51,5.55) | 5.53(5.50,5.55) | 5.54(5.52,5.57) | 0.39 |
| TG, mmol/L | 1.51(1.46,1.56) | 1.49(1.45,1.53) | 1.51(1.45,1.57) | 1.39(1.35,1.43) | <0.001*** |
| TC, mmol/L | 4.99(4.97,5.02) | 5.05(5.02,5.08) | 5.10(5.06,5.13) | 5.08(5.05,5.11) | <0.001*** |
| HDL-C, mmol/L | 1.28(1.27,1.30) | 1.34(1.33,1.35) | 1.38(1.36,1.39) | 1.46(1.44,1.47) | <0.001*** |
| LDL-C, mmol/L | 2.99(2.96,3.02) | 3.01(2.97,3.04) | 3.00(2.97,3.04) | 2.98(2.94,3.01) | 0.55 |
| CRP, mg/dl | 0.43(0.41,0.46) | 0.42(0.39,0.44) | 0.37(0.35,0.39) | 0.34(0.32,0.36) | <0.001*** |

Data of cardiometabolic indexes are presented as weighted mean [95% CI]. HEI-2015, healthy eating index-2015; FBG, fasting blood glucose; FBI, fasting blood insulin; HOMA-IR, homeostasis model assessment to evaluate insulin resistance; HbA1c, glycated hemoglobin; TG, triglyceride; TC, total cholesterol; HDL-C, high-density lipoprotein cholesterol; LDL-C, low-density lipoprotein cholesterol; CRP, C-reactive protein. * *P* value <0.05, ** *P* value <0.01, *** *P* value <0.001
